# Adaptive physics-informed trajectory reconstruction exploiting driver behavior and car dynamics

**DOI:** 10.1038/s41598-023-28202-1

**Published:** 2023-01-20

**Authors:** Michail A. Makridis, Anastasios Kouvelas

**Affiliations:** Department of Civil, Environmental and Geomatic Engineering, ETH, 8093 Zurich, Switzerland

**Keywords:** Civil engineering, Sustainability

## Abstract

As more and more trajectory data become available, their analysis creates unprecedented opportunities for traffic flow investigations. However, observed physical quantities like speed or acceleration are often measured having unrealistic values. Furthermore, observation devices have different hardware and software specifications leading to heterogeneity in noise levels and limiting the efficiency of trajectory reconstruction methods. Typical strategies prune, smooth, or locally modify vehicle trajectories to infer physically plausible quantities. The filtering strength is usually heuristic. Once the physical quantities reach plausible values, additional improvement is impossible without ground truth data. This paper proposes an adaptive physics-informed trajectory reconstruction framework that iteratively detects the optimal filtering magnitude, minimizing local acceleration variance under stable conditions and ensuring compatibility with feasible vehicle acceleration dynamics and common driver behavior characteristics. Assessment is performed using both synthetic and real-world data. Results show a significant reduction in the speed error and invariability of the framework to different data acquisition devices. The last contribution enables the objective comparison between drivers with different sensing equipment.

## Introduction

Technological advancements in road transport sensing technologies, driver assistance systems, and traffic-vehicle monitoring systems generate detailed trajectory data that produce unique insights on different topics, i.e., driver dynamics understanding, emissions and fuel consumption estimations, traffic and vehicle safety investigations, traffic management solutions, and maneuver monitoring. Usually, trajectory reconstruction is performed through pre-processing for outlier detection and further signal filtering. However, reconstructing different signals that might differ in noise, frequency, or sensor quality is challenging using a common strategy. Trajectory data are pruned, smoothed, or locally adjusted using heuristics, aiming for results that reflect physically compatible quantities.

There is a wide range of data collection and sensing technologies such as drones, cameras, Global Positioning Systems (GPS), cellular data, Bluetooth data, and others. In principle, vehicle trajectory monitoring involves regularly observing the vehicle’s position at discrete points in time. Then usually, speed and acceleration are derived from the fusion of position measurement with other sensors (e.g., accelerometers, gyroscopes, radars) or direct derivation of positions and speeds, respectively. Different data acquisition devices employ different characteristics (accuracy, noise, sampling frequency), and raw data, in most cases, if not all, need post-processing.

Vehicle trajectory extraction, reconstruction, filtering, or smoothing are closely related and have attracted research interest more than half a century ago with the seminal works of^1^ (i.e., Wiener filter), and^2^ (i.e., Rauch-Tung-Striebel smoother). It is an important topic that can enable investigations on various other topics, e.g., emissions or energy demand^3,4^, traffic dynamics^5–8^, modeling and control^9–11^, control^11^, connected and automated vehicles and driver understanding^12,13^, while this list is not exhaustive.

At the same time, more and more datasets with experimental observations have become publicly available, and nowadays, it becomes easier to organize experimental campaigns and study complex phenomena empirically. Filtering techniques are applied to the raw data to remove noise and obtain quality measurements. In the pNEUMA dataset^14^, the post-processing is performed by an Advanced Kalman filter without further information on the model or parameters. In the HighHD dataset^15^, the RTS algorithm^2^ is applied for post-processing. The OpenACC dataset^16^ contains different experiments using either local regression or moving average, while other works^17,18^ use moving average for trajectory filtering.

Recently, Toledo et al.^19^ used weighted local regression to derive a smooth time-continuous trajectory function, thus proposing a technique independent of the sampling frequency. Different window sizes and polynomial orders are tested, observing that fluctuations decrease with an increase in the window size, but increase with higher orders of the fitted polynomial. In another interesting work^20^, the authors have proposed a four-step approach, first removing extreme positional errors, then applying a low-pass filter, third reconstructing the trajectory locally, and finally, removing the residual noise with another low-pass filter. Furthermore, within the broader family of frequency-related techniques, Fard et al.^21^ have proposed a robust wavelet-based two-step method for vehicle trajectory reconstruction. Results have been presented for the well-known NGSIM database, i.e., the Next Generation SIMulation program of the US Department of Transportation in 2002. Additionally, Punzo et al.^22^ have proposed a Kalman-based filtering technique to reconstruct car-following trajectories and maintain platoon consistency. Finally, working on trajectory extraction from unmanned aerial vehicles, Chet et al.^23^ have proposed a novel methodological framework for automatic and accurate vehicle trajectory extraction from aerial videos.

Estimating the inherent noise in experimental data is challenging, although some data acquisition devices provide such analysis. Validation of any applied methodology is impossible without ground-truth information^21^. A common approach is to focus on tackling irregularities, i.e., abnormalities that can be detected with high certainty to comply with comfortable and maximum acceleration standards. For example, an observation of an extreme acceleration (e.g., 10 $[\hbox {m/s}^2]$) or an extreme deceleration during car-following (e.g. –10 $[\hbox {m/s}^2]$) are unarguably unrealistic values. Consequently, anything below (above) that observed acceleration (deceleration) is closer to the real value. The above example can also be applied to movements or speeds, but due to differentiation, such irregularities are more prominent in acceleration. The second indication of abnormal patterns is the high local acceleration variance, even under stable conditions^19–21^.

Without ground-truth data, the trajectories are filtered to a point where the inferred accelerations, speeds, and movements converge to values that seem reasonable from physics and experience points of view. Usually, there are commonly adopted parameter values per methodology to adjust the filtering strength^24^. Additionally, Montanino et al.^20^ validate their approach from a platoon consistency viewpoint, which is also important from a traffic flow perspective.

The vehicle’s power dynamics and general driving characteristics directly impact how a vehicle moves. This additional source of information, already exploited in modeling and simulation activities^25–27^, can be exploited in trajectory reconstruction. The synergistic combination of mathematical or physical models and data^28–32^ is the general idea and motivation behind the design of the proposed framework.

We know from vehicle power dynamics that the maximum vehicle acceleration can be modeled as a function of the vehicle’s speed. Consequently, acceleration values that seem perfectly normal at lower speeds are unrealistic at higher speeds^33^. Driving behavior (for the longitudinal direction) poses additional constraints in the feasible domain of observed accelerations. Real-world data show that human drivers systematically exploit less than 50% of the vehicle’s acceleration capacity at any given speed^34^. Therefore, we argue that even if the resulting acceleration values seem feasible from a vehicle’s power perspective, they might rarely be observed in real-world measurements.

This paper proposes a novel approach that builds on the benefits of traditional techniques and exploits their performance in trajectory reconstruction by injecting additional information about vehicle dynamics and driver behavior (for the longitudinal direction) in a straightforward yet rigorous manner. We propose a threshold-free interactive approach that derives the filtering strength automatically. The main idea is to exploit model-based approaches and reconstruct vehicle trajectories to the point that derived speeds and accelerations not only make sense from a physics point of view but are compliant with the vast majority of the observed vehicles/fleet and drivers on road transport systems.

The framework employs an iterative procedure that includes three main components: (a) the Vehicle Dynamics Constraint process (*VDC*), (b) the Driver Dynamics Compliance process (*DDC*), and (c) the Noise Reduction process (*NR*). The first component models the observed vehicle’s acceleration capacity as a speed function. The *VDC* box constrains all accelerations outside this acceleration capacity space. The second component models the acceleration capacity of ordinary observed drivers as a speed function. The vehicle specifications that can be retrieved from online open databases are needed for the first two components, i.e., gearbox, maximum torque, mass, etc., to model the acceleration capacity functions. The acceleration capacity functions for the vehicle’s capability and the ordinary behavior of drivers are modeled using the MFC model, considering also observed findings in the literature^25,34^. For implementation details, we refer the reader to the corresponding paper and the publicly available library [https://pypi.org/project/co2mpas-driver/]. The third component filters the signal with a predetermined filtering method. The filtering strength (also reported in this work as magnitude) is automatically detected through an iterative process that monitors and minimizes the local acceleration variance^29^ and is described in detail in the methodological section. It should be noted that the entire adaptive process is automatic and invariant to the quality of the observed data.

**Figure 1:**
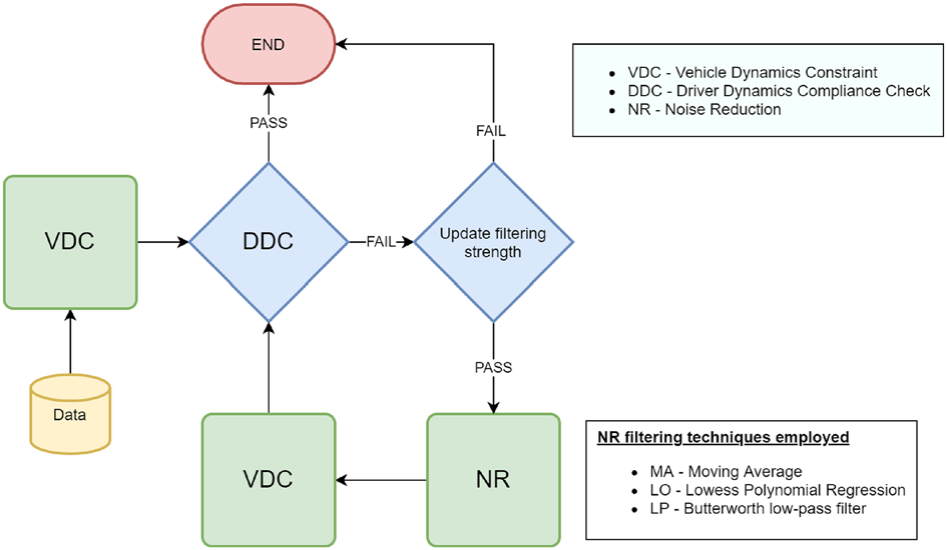
Flowchart for the proposed physics-informed framework.

The framework’s assessment is performed by utilizing synthetic and real-world data. Highly accurate differential GPS observations are used as reference trajectories, i.e., ground truth. Five levels of Gaussian noise are added, leading to 5 noisy trajectory datasets. Then, the proposed approach is tested on its ability to reconstruct the reference trajectories. Additionally, two mobile devices obtain real-world data on the same vehicle trajectory. The two devices have very different specifications resulting in visually different observations. The proposed methodology is applied to each signal separately and reduces the signal differences to the extent that the framework can be considered invariant to the acquisition device. Therefore, the results of the real-world campaign are promising for comparative investigations between different drivers. Figure 1 illustrates a high-level summary of the proposed framework.

## Results

Synthetic and real-world data are used to assess the robustness of the solution. Synthetic noisy data are generated by adding noise to observations obtained using highly accurate differential GPS devices (the AstaZero campaign of the OpenACC dataset^16^). Data from this campaign are used as a reference, i.e., ground truth. Five noisy synthetic trajectories are generated, assuming the reference data are noise-free. These trajectories, namely *N1, N2, N3, N4, N5*, have zero mean Gaussian noise and increasing standard deviation from 0.05 to 0.25 [m/s] at frequency of 10 [Hz]. The noise levels reflect the error estimates given by commercial data acquisition systems, such as U-blox. Three state-of-the-art filtering techniques are tested within the framework in the *NR* component (see Fig. 1), i.e., Moving Average (*MA*), Lowess Polynomial Regression algorithm (*LO*), and Butterworth filter (*LP*), while other similar filtering approaches can also be hosted. We compare the performance of each method when used independently with parameters suggested in the literature versus the same method when employed within the proposed framework, where the filtering strength is adaptively adjusted. When the methods are used independently, fixed parameters are assumed, a time window of 3s for *MA*, a 7-step window for *LO*, and a cut-off frequency of 0.75 [Hz] for *LP* (see^20^ for details). When the above techniques are used inside the proposed framework, their notation is *pMA*, *pLO*, and *pLP*, respectively, and their filtering magnitudes (window, steps, or cut-off frequencies) are inferred adaptively. Additional insights that demonstrate the robustness of the proposed methodology in comparison to more sophisticated methodologies for trajectory reconstruction (here used the Fard et al. technique^21^) are given in the Appendix document.

**Figure 2:**
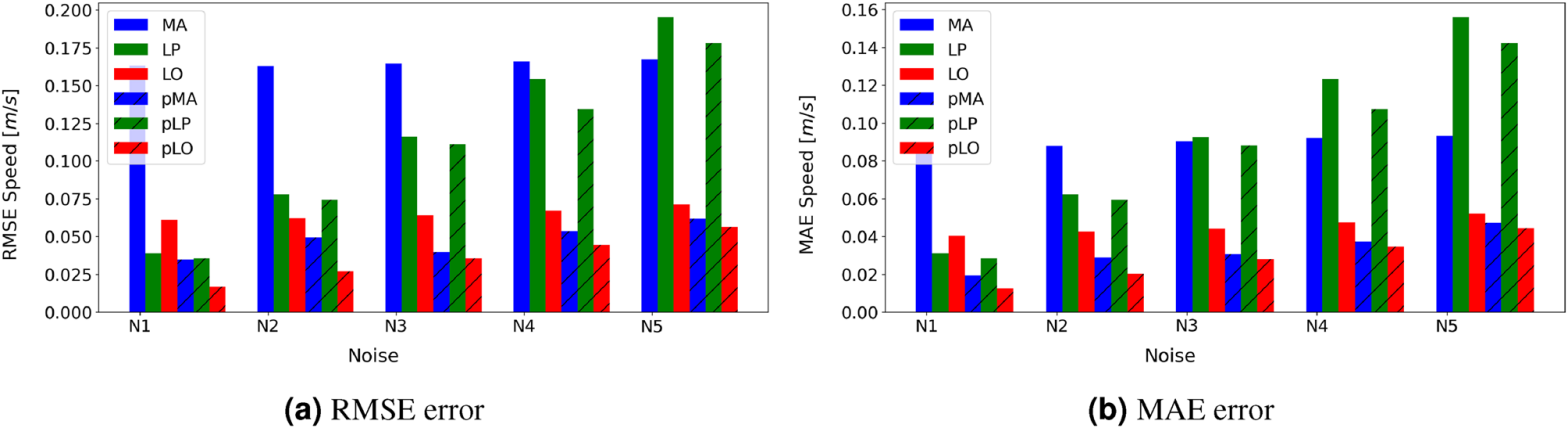
The RMSE and MAE errors for the three reconstruction methods with *pMA, pLP, pLO* and without (*MA, LP, LO*) the proposed framework.

Figure 2a,b illustrate the $I_{\text{RMSE}}$ and $I_{\text{MAE}}$ indicators for each scenario, that is, the root mean square error and mean absolute error of speed indices, as they are described in the methodology. The incorporation of each one of the three tested methods (*MA, LP, LO*) within the proposed framework (*pMA, pLP, pLO*) leads to a significantly improved reconstruction. Higher noise levels correspond to higher errors in comparison to the ground truth. The *LO and pLO* methods are the best performing and independent of the data frequency, which is a considerable advantage^19^. The *LO* performs well even with fixed window size, and the efficiency is comparable to the more complex counterparts of *pMA* and *pLO*. Within the proposed framework (*pLP*), the error decreases drastically, and it outperforms all the other methods compared to the other methods. The *MA and pMA* are fast, overall reliable, and almost independent of the noise levels, while *LP and pLP* are not recommended for this task.

The proposed framework considers the specifications of the vehicle known. However, this is not always possible in real-world campaigns. Consequently, for large-scale application of the proposed framework, average vehicle dynamics^25^ using representative vehicles from Euro Car Segments, as they are defined by European Commission policy^35^ can be employed. Alternatively, a more generic vehicle dynamics model^26^ with average vehicle specifications can also substitute the proposed MFC.

Table 1 shows the inferred optimal window size and cut-off frequency as computed adaptively by the proposed framework. The *MA* has been applied with fixed window, i.e., 3 [s], the *LO* with fixed steps, i.e., 7 (or 0.7 [s] at 10 [Hz] data), and *LP* with fixed frequency (0.75 [Hz]), which are commonly suggested values in the literature.

**Table 1:** Optimal filtering strength for each technique with the proposed framework.

	N1	N2	N3	N4	N5
*pMA*	1.2 [s]	1.5 [s]	0.8 [s]	1.3 [s]	1.1 [s]
*pLO*	25	31	29	31	37
*pLP*	0.65 [Hz]	0.7 [Hz]	0.7 [Hz]	0.60 [Hz]	0.65 [Hz]

For *pMA* and *pLO* methods, the first number corresponds to the number of observed points, while the number in parenthesis corresponds to its equivalent in terms of time.

The application of the proposed adaptive approach improves the efficiency of each method with ordinary filtering strength by up to 80% in terms of error reduction. However, aggregated indicators do not give insights about the resulting microscopic dynamics after the trajectory reconstruction, essential for accurate traffic flow and energy-related conclusions^4^.

**Figure 3:**
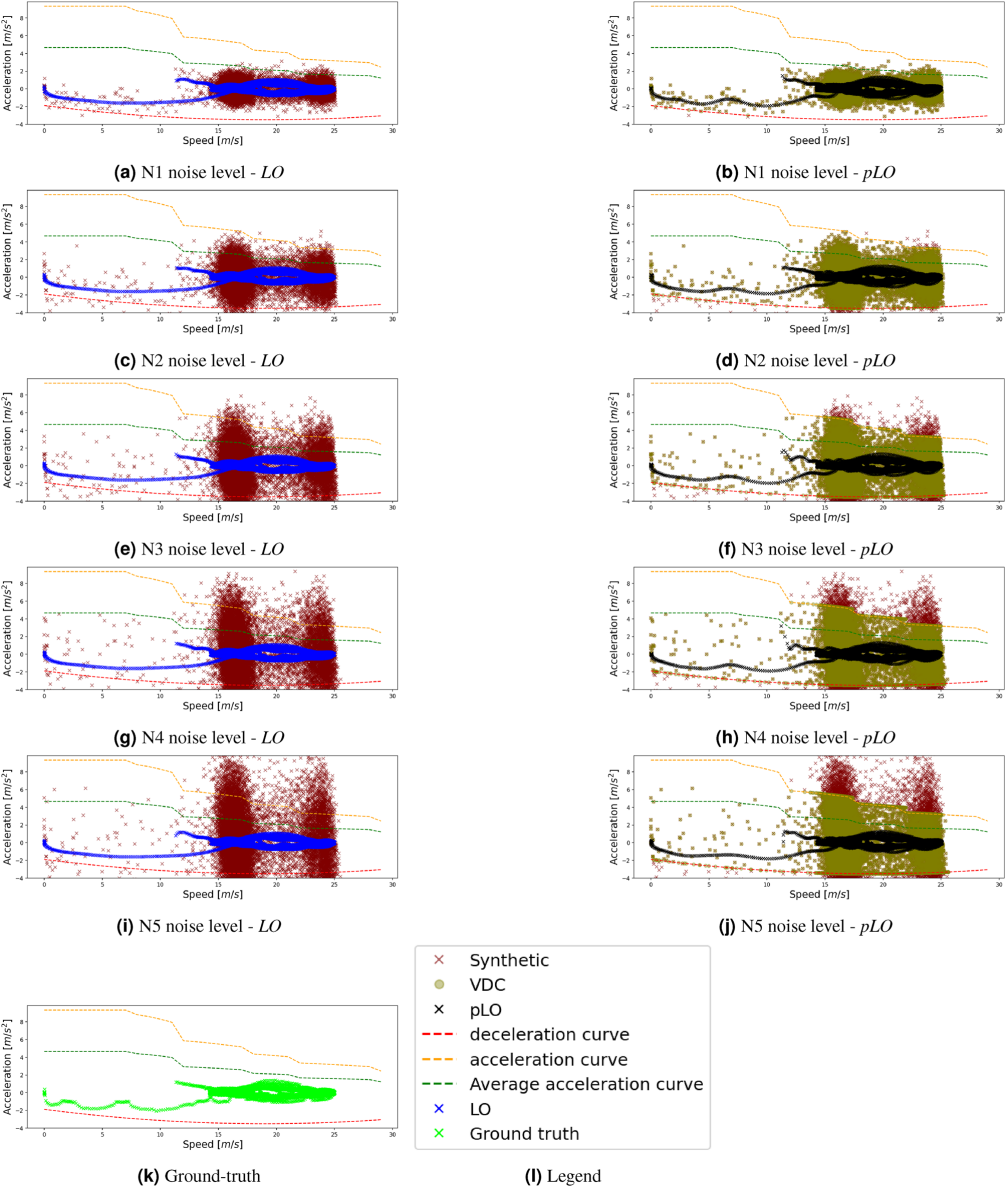
Campaign 1: Comparative results between the Lowess Polynomial Regression with and without the proposed framework for different noise levels.

Insights on the reconstructed microscopic dynamics follow in Fig. 3. Figure 3a,c,e,g,i illustrate the acceleration over speed values for each synthetic dataset *N1, N2, N3, N4, N5* respectively (red dots). The blue dots correspond to the filtered acceleration speed values after the application of the *LO* method. Figure 3b,d,f,h,j show results with the proposed framework. The red dots correspond to the synthetic datasets, the green dots to the corrections applied by the *VDC* component, and the black dots are the output of the proposed framework with the *pLO* method. Figure 3k shows the reference data, while Fig. 3l provides the legend for all sub-figures. The comparison of Fig. 3a,j with Fig. 3k demonstrates some interesting findings regarding the ability of each method to capture the observed acceleration dynamics.

An oscillation of acceleration values for speeds between 0 and 15 [m/s] is prominent in the ground truth data. This oscillation is mostly filtered by the individual *LO* application, while it is still observable in the results of the proposed physics-informed framework, i.e., *pLO*. Furthermore, the main body of acceleration observations between speeds 15 [m/s] and 25 [m/s] for the proposed technique is visually close to the ground truth data. The *LO* method homogenizes this area, resulting in smoother but distorted dynamics compared to ground truth data. The results show the efficiency of the proposed framework, *pLO* even for high noise levels as in scenarios N3–N5.

The second campaign took place in Greece. A vehicle’s trajectory is observed with two smartphones (S1 and S2), differing significantly in price, operating systems, and specifications. S1 costs half the price of S2, has Android, while S2 iOS, and records with an average rate of 1s (min/max: 0.04 [s]/2.89 [s]), while S2 records with the same frequency on average, i.e. 1s but different variance (min/max: 0.95 [s], 2.07 [s]).

Both signals are re-sampled to 1 [Hz] using linear interpolation. A lack of synchronization is most probably due to the internal clock of each device. Cross-correlation is used to estimate the time lag between the signals^5^.

**Figure 4:**
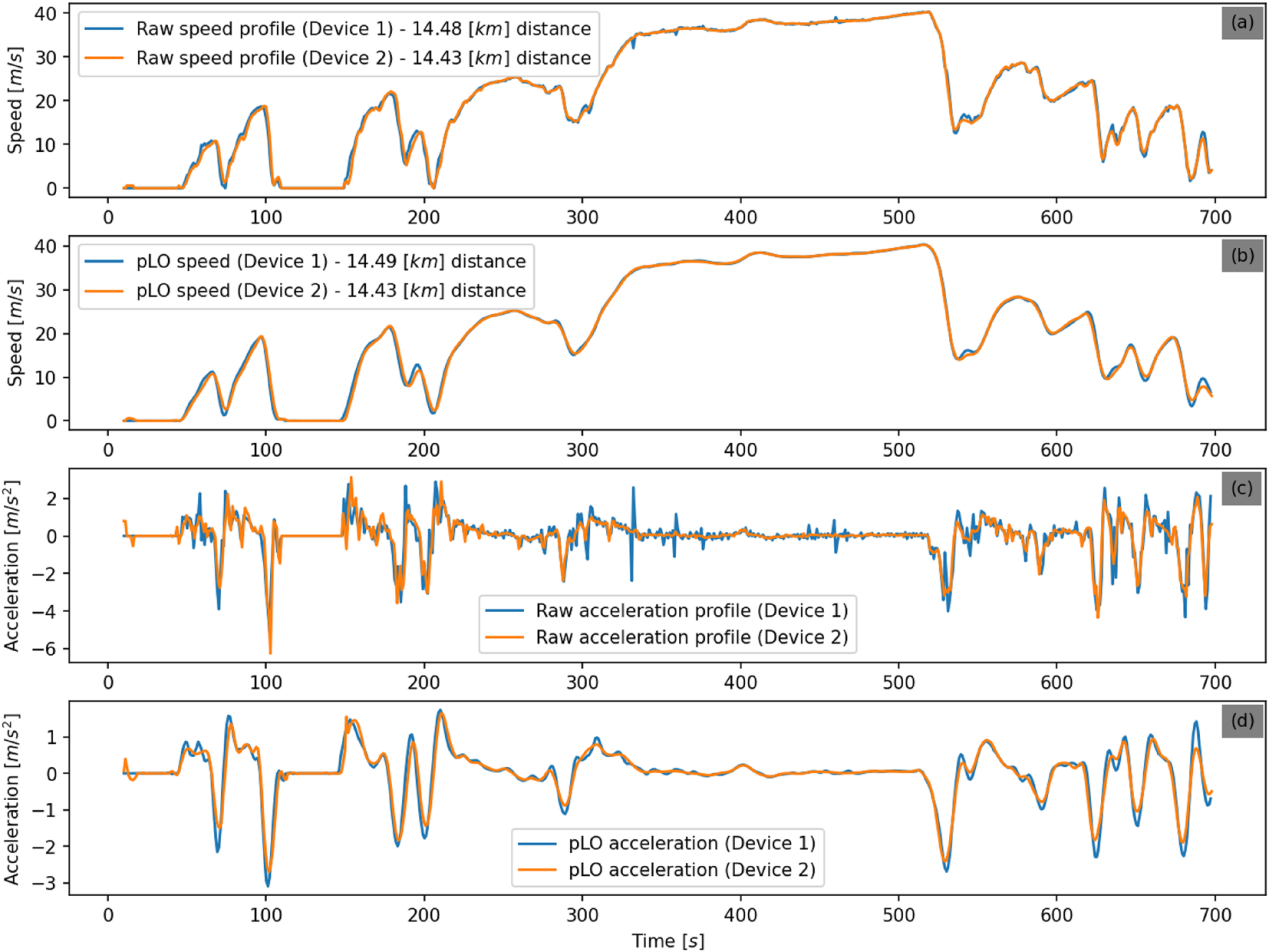
Campaign 2: The speed and acceleration profiles for the same trajectory with two devices, before and after the application of *pLO* method.

Figure 4 shows the speed and acceleration profiles per measurement device before and after the application of *pLO* method, which was the most efficient according to the results of the first campaign. The observed profiles are shifted in time, as mentioned above, to become synchronized. Both smartphones record the same vehicle trajectory (being with the driver), and the observed speed profiles are shown in the sub-figure Fig. 4a. Then the *pLO* is applied to both speed profiles, and the result is illustrated in the sub-figure Fig. 4b. Visually inspection reveals that most of the differences between the two signals have been smoothed, and it is obvious that they refer to the same measured trajectory. Going one order up, the acceleration observations are shown in the sub-figure Fig. 4c. Here, the differences between the observations of the two devices are more prominent. After the application of the proposed framework (*pLO*), the generated acceleration signals become very similar, pointing to the same measurements; see the sub-figure Fig. 4d. This result is very desirable in trajectory reconstruction. It can provide reliable results for several applications involving user comparisons concerning driving behaviors, fuel consumption profiles, and others.

Table 2 provides a quantitative evaluation for the second campaign, comparing the mean absolute error (MAE) and the median absolute deviation (MAD) between the two observed signals before and after the application of the proposed methodology. The comparative results refer to speed and acceleration values. In terms of speed, the improvement by the adaptive methodology is around 22.7% and 18.2% for MAE and MAD. The corresponding values for acceleration are even higher, i.e., 69.7% and 72.5% for MAE and MAD.

**Table 2:** Mean absolute error and median absolute deviation on speed and acceleration between the two observed signals before and after the application of *pLO* method.

	MAE before	MAE after	MAD before	MAD after
Speed [m/s]	0.44	0.34	0.22	0.18
Acceleration $[\hbox {m/s}^2]$	0.33	0.1	0.16	0.044

## Discussion

The vehicular and driving behaviors are known in traffic engineering as perhaps the most significant factors for raising complexity and leading to the appearance of non-linear phenomena in road transport systems. Until recently, experimental observations regarding detailed vehicle trajectory data have been scarce. During the last decade, technological advances and cost reductions in sensors enabled the organization of large and complex experimental campaigns. Such datasets, many of which are publicly available, provide invaluable insights into traffic engineering topics, provided they have a low error in observations.

There is no standardized protocol for designing and executing such experiments. Therefore, each experiment is performed with a different acquisition device, e.g., smartphones, U-blox, OXTS, etc. Such devices have various hardware and software specifications; therefore, the output signals are different even under the same conditions.

Noise removal in the measurements is often performed with arbitrarily parametrized filtering approaches, leading to questionable results since no ground-truth reference is available. The main idea is to remove by thresholding obvious outliers, i.e., values with no physical meaning, and then smooth the observation series globally or locally (on time or frequency domain) to derive a set of plausible measurements. The above strategy is problematic by design since vehicular and driver dynamics are nonlinear and observations with different devices need custom parametrization that is not obvious.

Exploiting the recent modeling advances in longitudinal vehicle dynamics simulation and driver behavior, the current methodology explicitly considers these two dimensions for trajectory reconstruction. The framework design is flexible and model-agnostic. A convergence process initiates, and an iterative process automatically adjusts the filtering strength based on vehicle and driver dynamic constraints.

Results on synthetic data generated based on low-error reference observations show that the proposed framework remarkably removes outliers and noise, maximizing the efficiency of the employed filtering strategy by automatically parametrizing the filtering strength. Furthermore, real-world observations on the same trajectory with two different devices show significant differences between measurements. The proposed framework is applied in both measured trajectories that visually demonstrate remarkable resemblance afterward, which is normal as they refer to the same experiment.

The above results are unique among trajectory reconstruction methodologies. Compliance with realistic nonlinear vehicle and driver dynamics ensures a fair comparison of observed trajectories and behaviors in various topics such as traffic flow, safety, driver identification, etc. Furthermore, the possibility to identify the same experiment from observations with different noise levels facilitates cross-driver comparisons providing reliable insights into topics such as energy consumption, driver aggressiveness, etc.

The main assumption of this framework is that the vehicle specifications, i.e., gearbox, gear ratio, mass, maximum torque, etc., are considered known for each vehicle trajectory^25^. This assumption is quite heavy because this information is not always given, especially in large complex experimental campaigns. However, it is shown that the vehicle dynamics can be efficiently clustered based on some main specifications^35^. The vehicles in the same cluster demonstrate similar dynamics, so no vehicle-specific details are necessary. Alternatively, there are more generic models than MFC, see for example^26^, that use average vehicle specifications and are much more flexible to be used within the proposed framework. It is worth noting that the dynamics of electric powertrains differ significantly from vehicles with Internal Combustion Engines, as the first can offer much higher torque from very low speeds. Therefore a different modeling approach should be used for electric vehicles. An implementation of the MFC for electric powertrains is also publicly available^36^. Finally, an extension or adoption of the proposed framework to capture both lateral and longitudinal dynamics would be interesting as a future work^37^.

## Methods

A physics-informed adaptive framework for trajectory reconstruction is proposed in this work. The algorithm employs three main components, as illustrated in Fig. 1: (a) the Vehicle Dynamics Constraint (*VDC*) process, (b) Driver Dynamics Compliance (*DDC*) process, and (c) Noise Reduction (*NR*) process. The next subsections describe these components individually, while the last subsection here presents the experimental campaigns and scenarios used for assessing and validating the approach, as well as the performance indicators.

**Figure 5:**
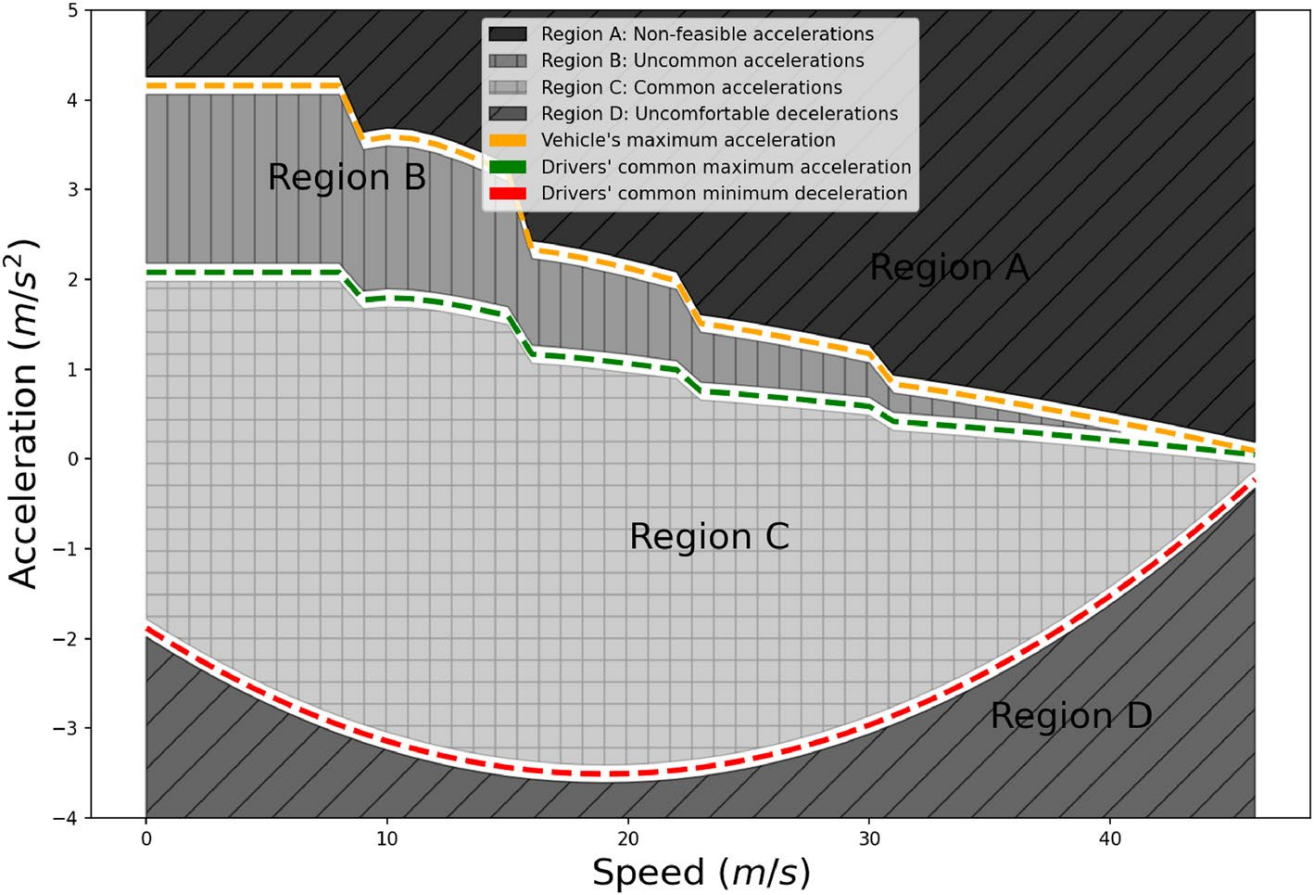
The acceleration-speed domain for a given vehicle and an average driver as computed in^25,36^.

Before discussing the individual components, it is important to elaborate on how vehicle and driver dynamics are modeled and considered in this approach. Figure 5 aims to clarify vehicle and driver dynamics from a modeling perspective in an illustrative way. Using a vehicle’s specifications (powertrain, engine power, mass, etc.), MFC microsimulation model^25^, used in this framework, describes the vehicle’s continuous acceleration capacity function, namely $a_p(v)$. This function returns the maximum possible acceleration for this specific vehicle at any given speed *v*. Figure 5 depicts this with the orange line.

Similarly, we compute the continuous minimum comfortable deceleration curve^36^, namely $d_p(v)$ (non-safety-critical situations). This function provides the minimum possible acceleration for this specific vehicle and a given speed *v*. Figure 5 depicts this with the red line.

These two curves inscribe our vehicle power domain. In other words, assuming that the employed vehicle dynamics model is accurate, any real acceleration value for a given speed that falls outside this domain is considered unrealistic (infeasible). Although the results in this work refer to specific vehicle models, it should be noted that the proposed methodology can be expanded for vehicle classes, according to Euro Car Segments, defined by the European Commission^38^, without significant loss of precision.

Furthermore, driver behavior observations reveal that most drivers do not even approach the acceleration capacity of their vehicle for any given speed. Recently, Makridis et al.^34^ conducted an unbiased experiment with 20 individuals driving freely (without any given instructions) across Europe the same vehicle over one year. The authors proposed a methodology to characterize the aggressiveness of these drivers and possibly cluster them in groups. One of the conclusions in that work has been that in all observations, maximum observed acceleration never exceeded 50% of the vehicle’s acceleration capacity for that given speed. Based on this conclusion, we compute the continuous acceleration capacity function of the driver, namely $a_d(v)$ as follows:

1 $$\begin{aligned} a_d(v) = d \cdot a_p(v) \end{aligned}$$

where *v* is a given speed and *d* is a scaling parameter. As mentioned above, in the current work, this parameter is fixed to 0.5, which is the recommended value for common-purpose car-following/driving experiments. In particular cases, such as experiments with free-flow accelerations to racing events, *d* should be tuned to values much closer to 1. This function provides the maximum estimated acceleration for most drivers and a given speed *v*. Figure 5 depicts this threshold with the green line.

Using the above three functions, $a_p(v)$, $d_p(v)$ and $a_d(v)$, we partition the acceleration over the speed domain for all possible observations in four main regions (Fig. 5) as follows:

- Region A: This set, $R_A$, includes all possible acceleration values for a given speed that the vehicle can not exploit. Any observation inside Region A is considered an outlier and should be box-constrained to a realistic value.
- Region B: This set, $R_B$, includes all possible acceleration values for a given speed that the vehicle can exploit but are considered not highly probable for common (ordinary) driving conditions. Any observation inside Region B is considered a noisy measurement and therefore indicates the necessity for noise reduction.
- Region C: This set, $R_C$, includes all possible acceleration values for a given speed that the vehicle can exploit and can potentially correspond to ordinary driving conditions. Any observation that lies inside Region C is acceptable as an accurate measurement. Therefore, there is no way to assess it and evaluate if the measurement corresponds to the actual value or not.
- Region D: This set, $R_D$, includes all possible acceleration values for a given speed outside a driver’s comfortable deceleration values. Any observation inside Region D is considered an outlier and should be box-constrained to a realistic value.

In the context of the current application of reconstructing vehicle trajectories, we denote *x*(*t*) as the time series of position measurements for a given vehicle. Moreover, *s* is the time-step, and $f=1/s$ is the sampling frequency. Correspondingly, speed and acceleration measurements are obtained with derivation, namely ${\dot{x}}(t)$ and $\ddot{x}(t)$. Additionally, if $N_A$, $N_B$, $N_C$, $N_D$ are the observations that fall in the corresponding regions $R_A$, $R_B$, $R_C$ and $R_D$, respectively, then, $N_A + N_B + N_C + N_D = N$. Finally, the present study can be employed for electric vehicles that demonstrate different power characteristics by utilizing the corresponding version of the MFC model^36^.

### Vehicle dynamics constraint

The goal of *VDC* is to ensure that all the observations in regions $R_A$ and $R_B$ are box-constrained to physically possible values, i.e., orange and red lines at the boundaries of regions $R_B$ and $R_C$ in Fig. 5. An advantage of the proposed framework over existing works is that it constrains the outliers nonlinearly. It is a common phenomenon in experiments to produce outliers, values that do not make any sense from the point of view of physics. Such values may appear due to different factors, such as weather conditions, road geometry, sensor errors, malfunctions, and others. Hard thresholding is commonly used in the literature toward this scope, i.e., removing accelerations above 5 $[\hbox {m/s}^2]$ and below $-8\;[\hbox {m/s}^2]$. However, the application of a horizontal threshold is not efficient. For instance, an acceleration of 3 $[\hbox {m/s}^2]$ can be achieved by a vehicle under low speeds, but it is unrealistic for high speeds, i.e., over 100 [km/h]. Reliable data acquisition systems might capture only a few such observations, i.e., that lie in $R_A$ or $R_D$, as they could also have their built-in filtering techniques. However, when data are collected with low-accuracy devices (e.g., mobile phones), this process plays an essential role.

The resulted observations set that is constrained by vehicle dynamics is called $\ddot{x}_{\text{vdc}}$ and can be described as follows:

2 $$\begin{aligned} \ddot{x}_{\text{vdc}}(t) = \left\{ \begin{array}{ll} a_p({\dot{x}}(t)), &{} \text{ if }\;{\dot{x}}(t) \in R_A \\ d_p({\dot{x}}(t)), &{} \text{ if }\;{\dot{x}}(t) \in R_D \\ \ddot{x}(t), &{} \text{ otherwise } \end{array}\right. \end{aligned}$$

This process is applied iteratively inside the proposed methodology for increasing filtering strength, generating a new set of values at each iteration. Therefore, the paper uses an indicator *l* to describe the loop count wherever necessary.

### Driver dynamics compliance

The goal of *DDC* is to ensure that all observations respect the average driver behavior and automatically assess the filtering magnitude that will be applied by the noise reduction technique. Ultimately, the framework should work independently of the noise reduction methodology and its parameters related to the filtering strength. One of the main problems in traditional trajectory reconstruction techniques is the definition of granularity in the noise reduction processes. For example, in low-pass filtering, the cut-off frequency can vary depending on the noise levels in the raw data and/or sampling frequency. Similarly, in polynomial regression or moving average, the span of the window that will be used for smoothing can heavily distort the observed profile. The proposed compliance check focuses on ensuring the following requirements:

- (*RQ1*) All acceleration values correspond to common driver aggressiveness levels.
- (*RQ2*) The variance of sequential accelerations locally in time is close to the variance typically observed in high-accuracy data acquisition systems (e.g., Differential GPS).

The first requirement, *RQ1*, aims at ensuring that measurements correspond to typical drivers. Typical drivers do not exploit the full power and, thus, acceleration capacity of their vehicle^34^. The term aggressiveness is used within this work to characterize drivers that accelerate sharper than others, i.e., approaching the vehicle’s maximum acceleration for a given speed. According to Fig. 5, all observations should lie in the area below the orange line, i.e., the maximum possible acceleration of the vehicle at a given speed. In practice, most observations fall around the middle of the area inscribed by the orange and red lines (comfortable deceleration as a function of speed). Observations near the orange line or below the red line are rarely observed, indicating excessive aggressiveness in driving. In our opinion, this is something to be considered in trajectory analysis. Of course, it should be noted that for particular types of experiments (e.g., maximum acceleration from 0 to 100 km/h), this requirement should be relaxed, i.e., the green line in Fig. 5 should be defined closer to the orange one ($R_B$ area becomes smaller). This process validates the ratio $r_l=N_{C_l}/N$, which is the number of observations that fall inside $R_C$, for iteration *l*, over the total number of observations *N*. The ratio $r_l$ is parametrized for every iteration *l* because the number of values that fall in $R_C$ can differ in every iteration. According to this criterion, an acceptable dataset should contain only a few values outside region $R_C$. Therefore, this ratio needs to be lower than a fixed threshold, namely $d_{\text{th}}$ (here set to 0.05, or 5% of the observations).

The second requirement, *RQ2*, aims to monitor the acceleration signal’s local variations. Under real-world non-critical driving conditions, the local variance of accelerations should be relatively low. In the frequency domain, the above irregular pattern is often encountered in empirical observations due to noise, and therefore, low-pass filtered techniques are commonly used to mitigate this effect. Consequently, even if all observations are compatible with vehicle and driver dynamics, the magnitude of local acceleration variations should be low before the application of a smoothing technique and therefore plays a decisive role in the parametrization of the filter’s strength (i.e., window or cut-off frequency).

On the other hand, the amount of noise in a signal directly impacts the observed variations. Furthermore, each technique has different efficiency in alleviating these variations, which is not known beforehand. The proposed work estimates the parameters that impact the filter strength based on the above. Assuming that we have *N* acceleration observations $\ddot{x}(t)$, their local standard deviation $\sigma _{\ddot{x},l}(k)$, for iteration *l* around *k*, and a fixed window $w_{\text{var}}$ (set to 1.5 s) is computed as follows:

3 $$\begin{aligned} \sigma _{\ddot{x},l}(k) = \sqrt{\frac{1}{(2\cdot w_{\text{var}} + 1)}\sum _{i=k-w_{\text{var}}}^{k+w_{\text{var}}}\left( \ddot{x}(i) - \mu _k\right) ^2}, \end{aligned}$$

where

4 $$\mu _{k} = \sqrt {\frac{1}{{(2 \cdot w_{{\text{var} }} + 1)}}\sum\limits_{{i = k - w_{{\text{var} }} }}^{{k + w_{{\text{var} }} }} {\ddot{x}} (i)} .$$

Since the acceleration values change, the local standard deviation is parametrized per iteration, *l*. Furthermore, we consider as an indicator the median value of all observed local standard deviations, $\text{med}(\{\sigma _{\ddot{x},l}(k)\}, \forall k)$, namely $\text{med}(\sigma _{\ddot{x},l})$. If we consider that most of the time, the driver (either human or automated) aims at maintaining a constant speed, we can assume that $\text{med}(\sigma _{\ddot{x},l})$ will correspond to a prevalent value. However, standard noise directly impacts individual $\text{med}(\sigma _{\ddot{x},l})(k)$ values and consequently the global $\text{med}(\sigma _{\ddot{x},l})$ indicator. Furthermore, we define the following function:

5 $$\begin{aligned} f(w_l) = \frac{\big (\text{med}(\sigma _{\ddot{x},l-1}) - \text{med}(\sigma _{\ddot{x},l})\big )}{\text{med}(\sigma _{\ddot{x},l})}. \end{aligned}$$

**Figure 6:**
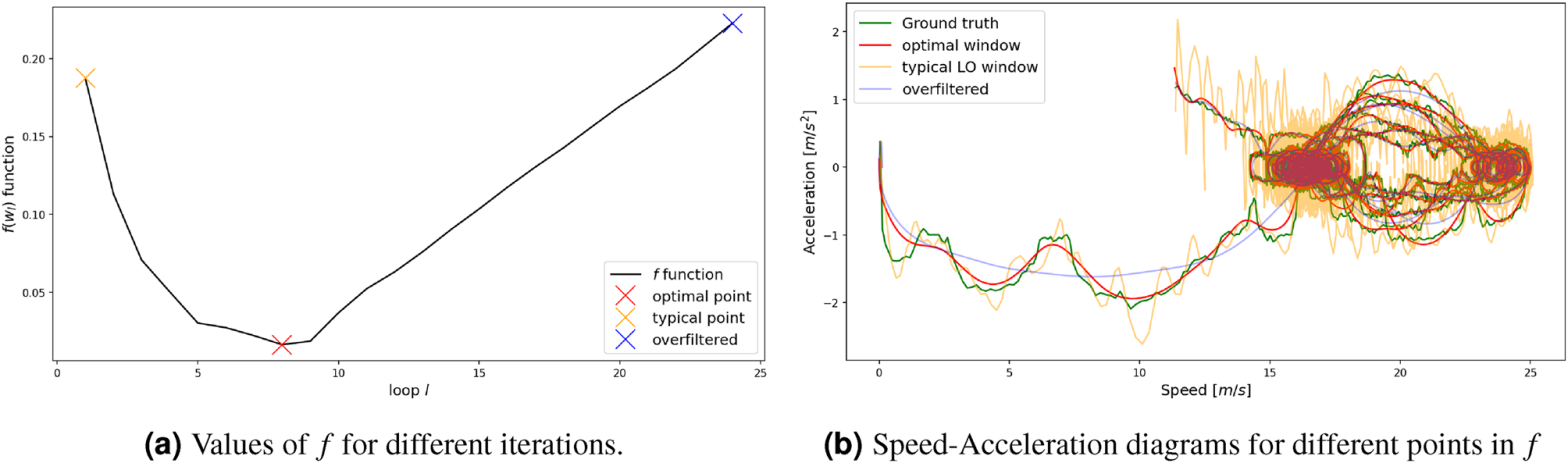
Example of *f* function over the number of iterations for the LO method with.

The idea behind function $f(w_l)$ is to automatically determine the filtering magnitude for the noise reduction component based on the reduction of the acceleration’s local variability. Specifically, increasing filtering strength at each iteration *l*, i.e., increasing the window/step size or lowering the cut-off frequency, $\sigma$ is expected to decrease respectively. At the same time, the rate of $\text{med}(\sigma _{\ddot{x},l})$, which in our discrete system is the normalized difference between $\text{med}(\sigma _{\ddot{x},l-1})$ and $\text{med}(\sigma _{\ddot{x},l})$, decreases as well, towards an optimal result corresponding to the applied technique. However, after passing the optimal threshold, we expect the function *f* to stop being monotonically decreasing and most likely fluctuate as the filter strength increases further (larger windows or lower cut-off frequencies).

An example of *f* function trajectory over iterations is shown in Fig. 6a. The trajectory’s three markers (red, yellow, and blue) correspond to the optimal, typical, and over-filtered window sizes. The idea is that until the red marker, we have high certainty that the noise decreases as the local standard deviation rate decreases. After that iteration, there is little knowledge about real noise reduction. Typical thresholds, depending on the dataset, might lead to a reasonable noise reduction level as was shown in Fig. 2a,b, but the final result depends on the dataset specifications (noise, frequency, etc.). The proposed technique ensures a satisfactory result and automatic inference of the filter’s magnitude. Figure 6b illustrates the acceleration over speed diagrams for the typical, optimal, and over-filtered points, showcasing the robustness of the proposed process.

Concretely, we set the filtering range threshold to the maximum filter range that can ensure that function *f* remains monotonically decreasing, that is, the size of the window or cut-off frequency $w_l$ for which $f(w_l)>f(w_{l-1})$. To this end, we have:

6 $$\begin{aligned} \text{RQ2} \left\{ \begin{array}{ll} \text{PASS}, &{} \text{ if }\;r_l \le d_{\text{th}} \wedge f(w_l) \ge f(w_{l-1}) \\ \text{FAIL}, &{} \text{ otherwise } \end{array}\right. \end{aligned}$$

### Noise reduction and trajectory reconstruction

Three well-known techniques have been employed for trajectory reconstruction and the assessment of the present methodology: a simple Moving Average (*MA*); Lowess Polynomial Regression (*LO*) (window parameter taken from^19^); and Butterworth (*LP*) (cut-off parameter taken from^20^). The window $w_{\text{MA}}$ for *MA* looks locally up to 15 observations, $w_{\text{MA}} \in \{1,2, \ldots , 15\}$. For *LO* the minimum window size $w_{\text{LO}}$ should be sufficient to adequately estimate the derived speeds and accelerations (see also^19^), thus $w_{\text{LO}} \in \{3, \ldots , 15\}$. The order was set to 6. Finally, a first-order Butterworth filter was implemented, and its cut-off frequency $f_{\text{LP}}$ decreases progressively down to 0.05 [Hz], $f_{\text{LP}} \in \{0.9, \ldots , 0.05\}$. We consider that the magnitude of a filtering technique increases as the output is smoother. Therefore, the maximum strength for *MA* and *LO* is with window size 15, while for *LP* with 0.05 cut-off frequency. Table 3 describes the proposed algorithm at a high level. The raw data are pre-processed with *VDC*, and then the algorithm controls if the specifications of *DDC* are met. If not, *NR* is applied, followed by the next iteration. This workflow applies iteratively with increasing noise reduction strength until either *DDC* conditions are met or the maximum *NR* strength is reached.

**Table 3:** High-level algorithmic workflow.

Initialization	
Step 1: Load raw data	
Step 2: Initialize filter strengths $w_{\text{MA}}$, $w_{\text{LO}}$ or $f_{\text{LP}}$	
Step 3: Apply *VDC*	
Step 4: Check *DDC*: If PASS GoTo Step 9; Else GoTo Step 5	
Step 5: Set $w_{\text{MA}}=w_{\text{MA}}+1$, $w_{\text{LO}}=w_{\text{LO}}+1$ or $f_{\text{LP}}=cf_{\text{LP}}-0.05$	
Step 6: Apply Noise Reduction (*MA*, *LO* or *LP*)	
Step 7: Apply *VDC*	
Step 8: Check *DDC*: If PASS or filter strength is maximum GoTo Step 9; Else GoTo Step 5	
Step 9: END	

### Performance assessment

The assessment of trajectory reconstruction methodologies is challenging when there is no ground truth data. In this paper, we employ two experimental campaigns with different campaigns to perform our assessment.

Campaign 1: The *AstaZero* experiments described in OpenACC dataset^16^ include low-noise position observations from multiple vehicles inside the AstaZero test track in Sweden. The measurements have low noise due to the differential GPS data acquisition equipment. This work uses around 25km of a vehicle trajectory in this test track. Because of the initial low noise levels, we consider this reference trajectory as ground truth. Gaussian noises of zero means and increasing standard deviation values are added to create noisy synthetic speed profiles. More specifically, 5 levels of standard deviation in [m/s] are considered in the results, $\{0.05, 0.1, 0.15, 0.2, 0.25\}$. The proposed framework is tested for the above three noise reduction methods and five noise levels. Moreover, comparisons are presented for the three noise reduction methods with parameters proposed in the literature.

For comparison indicators, the mean absolute error and root mean squared error on the speed profiles are used;

7 $$\begin{aligned} I_{\text{MAE}}= & {} \frac{1}{N}\sum _{i=1}^{N}|{\dot{x}}-\hat{{\dot{x}}} |\end{aligned}$$

8 $$\begin{aligned} I_{\text{RMSE}}= & {} \sqrt{\frac{1}{N}\sum _{i=1}^{N}({\dot{x}}-\hat{{\dot{x}}})^2} \end{aligned}$$

where *N* is the number of observations, ${\dot{x}}$ the ground-truth and $\hat{{\dot{x}}}$ the reconstructed trajectory.

Campaign 2: This campaign includes the observed trajectories recorded by the sensors of two smartphones via the Phyphox application^39^. Two different smartphone devices, one less and another more expensive, with Android and iOS operating systems, respectively, have been used during the same experiment. The idea is to assess the consistency of the proposed framework for different sensors and error levels on the same trajectory. We compute the mean absolute error and median absolute deviation between the two signals to provide quantitative assessment values.

## Supplementary Information

Supplementary Information.
