# Supplementary material for: Adaptive physics-informed trajectory reconstruction exploiting driver behavior and car dynamics
Source: Sci Rep. 2023 Jan 20;13:1121. doi: 10.1038/s41598-023-28202-1 (PMC9859820; doi:10.1038/s41598-023-28202-1)
Supplement: Supplementary file 1 — Supplementary Information. [file 41598_2023_28202_MOESM1_ESM.docx]

Appendix: Adaptive physics-informed trajectory reconstruction exploiting driver behavior and car dynamics

Michail A. Makridis^*^, Anastasios Kouvelas

*Institute for Transport Planning and Systems (IVT), ETH Zurich, 8093 Zurich, Switzerland
* corresponding author*

We would like to provide additional results with the Fard et al.^1^, a wavelet-based methodology for trajectory reconstruction, since this is currently considered state-of-the-art in this topic. Figure 1 shows that pLO captures more accurately the acceleration-speed dynamics even for the lowest noise level for the synthetic trajectory of Campaign 1.

| 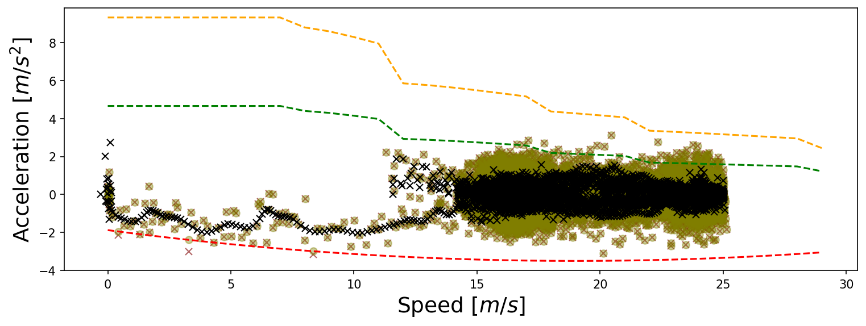 | 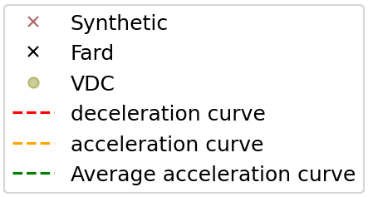 |
| --- | --- |
| (a) | (b) |
| Figure 1. Trajectory reconstruction with Fard et al.^1^ technique on the synthetic dataset *N1*. For the ground truth and the *pLO* results, we refer the reader to see Figure 3 in the original document. | |

Figure 2 shows that reconstruction results on the speed and acceleration profiles of both observations of the second campaign. The method successfully identifies and modifies outlier values in the speed and acceleration profiles. However, it does not produce similar results for the signals of the two devices, despite the fact that those signals refer to the same observed trajectory. This is more obvious in the result on the acceleration. For the results of the *pLO* method; we refer the reader to Figure 4 in the original document.

| 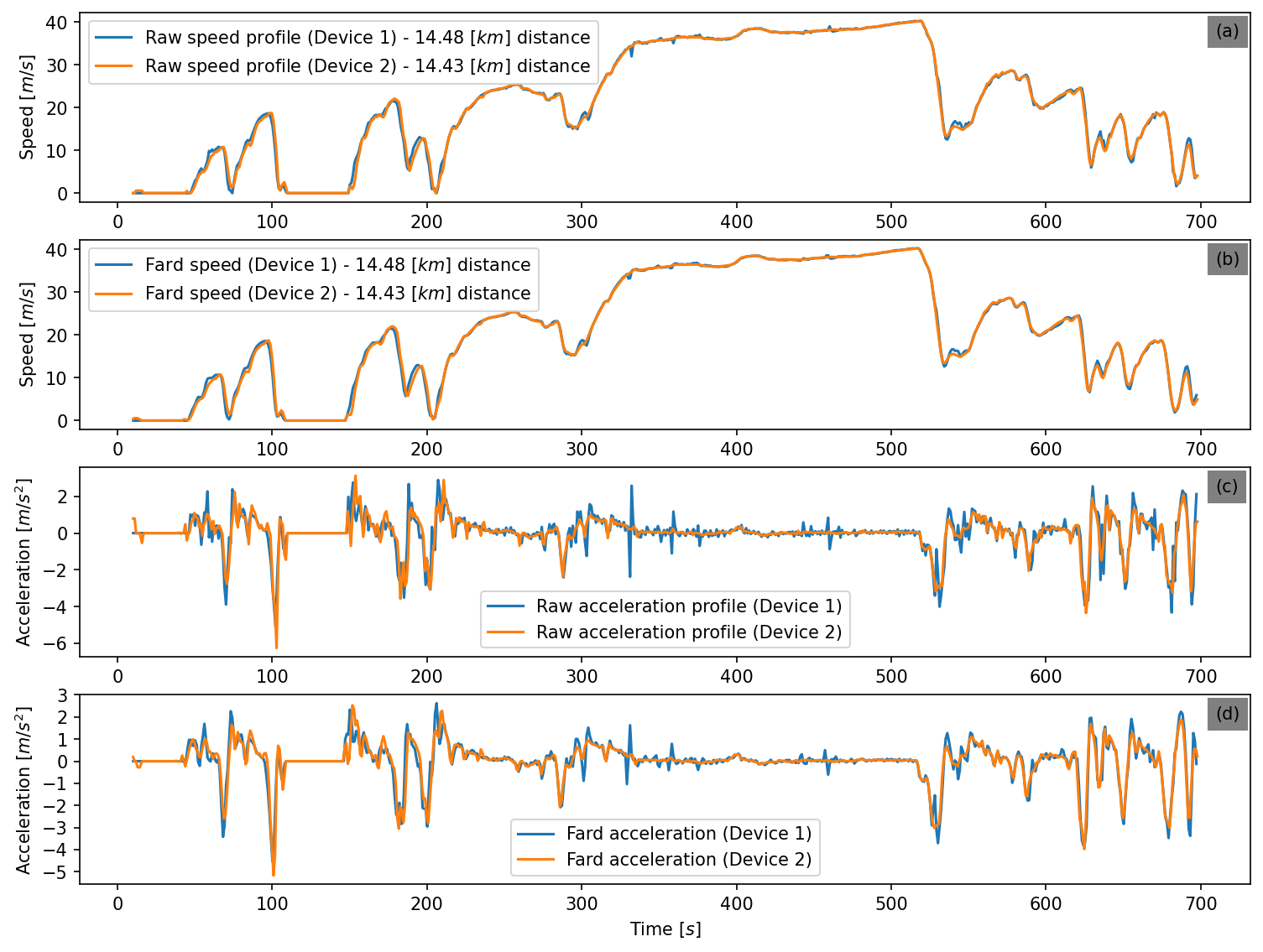 |
| --- |
| Figure 2. The speed and acceleration profiles for the same trajectory with two devices, before and after the application of the method proposed by Fard et al.^1^ for campaign 2. |

1. Fard, M. R., Shariat Mohaymany, A. & Shahri, M. A new methodology for vehicle trajectory reconstruction based on wavelet analysis. *Transportation Research Part C: Emerging Technologies* **74**, 150–167 (2017).
